# Weight and height z-scores improve after initiating ART among HIV-infected children in rural Zambia: a cohort study

**DOI:** 10.1186/1471-2334-11-54

**Published:** 2011-03-01

**Authors:** Catherine G Sutcliffe, Janneke H van Dijk, Bornface Munsanje, Francis Hamangaba, Pamela Sinywimaanzi, Philip E Thuma, William J Moss

**Affiliations:** 1 Department of Epidemiology, Bloomberg School of Public Health, Johns Hopkins University, Baltimore, MD, USA; 2 Macha Research Trust, Macha Hospital, Choma, Zambia

## Abstract

**Background:**

Deficits in growth observed in HIV-infected children in resource-poor settings can be reversed with antiretroviral treatment (ART). However, many of the studies have been conducted in urban areas with older pediatric populations. This study was undertaken to evaluate growth patterns after ART initiation in a young pediatric population in rural Zambia with a high prevalence of undernutrition.

**Methods:**

Between 2007 and 2009, 193 HIV-infected children were enrolled in a cohort study in Macha, Zambia. Children were evaluated every 3 months, at which time a questionnaire was administered, height and weight were measured, and blood specimens were collected. Weight- and height-for-age z-scores were constructed from WHO growth standards. All children receiving ART at enrollment or initiating ART during the study were included in this analysis. Linear mixed effects models were used to model trajectories of weight and height-for-age z-scores.

**Results:**

A high proportion of study children were underweight (59%) and stunted (72%) at treatment initiation. Improvements in both weight- and height-for-age z-scores were observed, with weight-for-age z-scores increasing during the first 6 months of treatment and then stabilizing, and height-for-age z-scores increasing consistently over time. Trajectories of weight-for-age z-scores differed by underweight status at treatment initiation, with children who were underweight experiencing greater increases in z-scores in the first 6 months of treatment. Trajectories of height-for-age z-scores differed by age, with children older than 5 years of age experiencing smaller increases over time.

**Conclusions:**

Some of the effects of HIV on growth were reversed with ART initiation, although a high proportion of children remained underweight and stunted after two years of treatment. Partnerships between treatment and nutrition programs should be explored so that HIV-infected children can receive optimal nutritional support.

## Background

Children in sub-Saharan Africa have high levels of undernutrition, exhibiting lower weight- and height-for-age than children in high resource settings. Both of these conditions are exacerbated by HIV infection [1-4], and can be used to determine disease status and monitor treatment response [5]. As implementation of antiretroviral treatment (ART) programs in sub-Saharan Africa has increased [6], many HIV-infected children are benefitting from treatment and are experiencing reductions in morbidity and mortality. Several studies have shown that many of the deficits in growth due to HIV infection are reversed with ART, with children exhibiting consistent improvements in weight-for-age [7-19]. Some studies, but not all [12,13,17], have also reported improvements in height-for-age [7-10,14,16,18,19]. Gains in weight have been found to correlate with treatment response [19].

Many of these studies have been conducted in urban areas, where food security tends to be higher and levels of undernutrition lower than in surrounding rural areas [20]. In addition, many of these studies were conducted in the first years of program implementation when the majority of children initiating ART were older [21]. Both age [11] and level of undernutrition at treatment initiation [7] have the potential to impact growth trajectories and the effect of ART. Consequently, this study was undertaken to evaluate growth after ART initiation in a young pediatric population in rural Zambia and identify characteristics at ART initiation that influence growth trajectories.

## Methods

### Study setting and population

The study was conducted at Macha Hospital in a rural area of Southern Province, Zambia. The study setting and population have been described in detail elsewhere [22]. Briefly, Macha Hospital is a district-level referral hospital administered by the Zambian Brethren in Christ Church. Since 2005, Macha Hospital has provided care to over 6000 HIV-infected adults and children through the Government of Zambia's antiretroviral treatment program, with additional support from the President's Emergency Plan for AIDS Relief (PEPFAR) through the non-governmental organization, AidsRelief.

Children with a positive HIV serologic test are referred to the clinic from voluntary counseling and testing programs, outpatient clinics and rural health centers. Early infant diagnosis has been available since February 2008. Clinical care is provided without charge by medical doctors and clinical officers, and adherence counseling by nurses and trained counselors. ART is initiated according to WHO guidelines [23,24]. The first-line antiretroviral treatment regimen consists of two nucleoside reverse transcriptase inhibitors (lamivudine (3TC) plus zidovudine (AZT) or stavudine (D4T) or abacavir (ABC)) and a non-nucleoside reverse transcriptase inhibitor (efavirenz (EFV) or nevirapine (NVP)). Pediatric and adult fixed dose combinations of D4T and 3TC are available, as well as of D4T, 3TC and NVP. High energy protein supplements are provided to underweight children.

### Study procedures

Beginning in September 2007, HIV-infected children younger than 16 years seeking HIV care were eligible for enrollment into a cohort study. Written informed consent was obtained from parents or guardians and assent was obtained from children 8-16 years of age. Children were evaluated at study visits approximately every three months, at which time a questionnaire was administered, the child was examined and a blood specimen was obtained. At each visit CD4^+ ^T-cell counts and percentages were measured using the Guava Easy CD4 system (Guava Technologies, Inc., Hayward, CA). During each physical examination, height and weight were measured. For children who missed study visits, home visits were attempted to ascertain their status. Information recorded before study enrollment was abstracted from medical records. The study was approved by the Ministry of Health in Zambia, the Research Ethics Committee of the University of Zambia and the Institutional Review Board of the Johns Hopkins Bloomberg School of Public Health.

For the present analysis, all children enrolled in the study and receiving ART between September 2007 and September 2009 were included. The study sample included children already receiving ART at enrollment and children initiating ART during the study period.

### Statistical analysis

Data were entered in duplicate using EpiInfo (Centers for Disease Control and Prevention) and analyses were conducted in STATA, version 9 (StataCorp LP, College Station, Texas). Weight-for-age z-scores (WAZ) among children younger than 10 years of age and height-for-age (HAZ) z-scores among all children were calculated based on the WHO growth standards [25], and children with z-scores below -2 were defined as underweight and stunted, respectively. Severe immunodeficiency was defined by CD4^+ ^T-cell percentage according to the WHO 2006 treatment guidelines [24].

WAZ and HAZ after ART initiation were assessed among children with at least one post-ART measure. Children were followed until they died, were lost to follow-up, or were administratively censored on September 30, 2009. Children who had not returned for at least 6 months were assumed lost to follow-up. For reporting outcomes at specific time points after ART initiation, measurements were aggregated to within 45 days. Treatment outcomes were evaluated using linear mixed effects models with random intercept, exchangeable correlation structure and robust standard error estimation. As changes in WAZ were not linear, a spline term was added at 7.5 months, the upper window around the 6-month measure. Covariates of interest included sex, orphan status, education of the primary caregiver, age, underweight, stunting and severe immunodeficiency at ART initiation. Covariates found to be (p < 0.10) or known to be associated with either outcome were included in the models. Differences in trajectories of WAZ and HAZ were assessed by each covariate of interest.

## Results

### Characteristics of the study population at study enrollment and ART initiation

Between September 2007 and 2009, 193 children received ART, with 67 entering the study already receiving ART and 126 initiating ART after study enrollment. Children receiving ART at study enrollment entered a median of 8.3 months (IQR: 2.3, 17.7) after initiating ART, while treatment-naïve children initiated ART a median of 2.0 months (IQR: 0.9, 6.0) after study enrollment. The median follow-up time in the study was 13.1 months (IQR: 5.1, 20.0). The median age was 3.0 years (IQR: 1.6, 6.9) at study enrollment and 51.3% were male (Table 1). The majority of children were cared for by a parent (77.5%) or grandparent (13.6%). Sixty-three percent of primary caregivers had no high school education and 9.5% of children were double orphans. Very few mothers (2.6%) had received drugs to prevent mother-to-child transmission. The median age at ART initiation was 2.9 years (IQR: 1.7, 6.8). The median WAZ and HAZ at ART initiation were -2.3 (IQR: -3.5, -1.4; 58.9% underweight) and -3.2 (IQR: -4.3, -1.9; 71.9% stunted), respectively. The median CD4^+ ^T-cell percentage at ART initiation was 16.3% (IQR: 11.5, 20.1; 59.8% severe immunodeficiency). Children who entered the study already receiving ART were significantly older and more likely to be male. In addition, they were significantly more likely to be underweight and have a lower CD4^+ ^T-cell percentage at ART initiation.

**Table 1:** Characteristics at study enrollment and ART initiation of HIV-infected children receiving antiretroviral therapy

	Total (n = 193)	Children receiving ART at study enrollment (n = 67)	Children starting ART during study period (n = 126)	p-value
***Study enrollment***				

Median age in years (IQR)	3.01 (1.62, 6.89)	4.25 (2.46, 8.61)	2.53 (1.27, 6.34)	0.0005
<1 yr	24 (12.4)	2 (3.0)	22 (17.5)	
1-1.9 yrs	38 (19.7)	8 (11.9)	30 (23.8)	
2-4.9 yrs	66 (34.2)	29 (43.3)	37 (29.4)	
5+ yrs	65 (33.7)	28 (41.8)	37 (29.4)	0.002
Male sex	99 (51.3)	43 (64.2)	56 (44.4)	0.009
Mother received PMTCT (%)	5 (2.6)	0 (0.0)	5 (4.0)	0.24
Vital status of parents (%)				
Both alive	135 (71.1)	39 (60.9)	96 (76.2)	
Mother died	19 (10.0)	7 (10.9)	12 (9.5)	
Father died	18 (9.5)	8 (12.5)	10 (7.9)	
Both died	18 (9.5)	10 (15.6)	8 (6.4)	0.10
Primary caregiver (%)				
Mother/father	148 (77.5)	48 (73.9)	100 (79.4)	
Grandparent	26 (13.6)	11 (16.9)	15 (11.9)	
Aunt/uncle	12 (6.3)	5 (7.7)	7 (5.6)	
Other	5 (2.6)	1 (1.5)	4 (3.2)	0.55
Education of primary caregiver (%)				
None	11 (6.3)	3 (5.3)	8 (6.8)	
Primary	100 (57.1)	31 (54.4)	69 (58.5)	
Secondary	62 (35.4)	23 (40.4)	39 (33.1)	
Higher	2 (1.1)	0 (0.0)	2 (1.7)	0.62

***ART initiation***				

Median age in years (IQR)	2.93 (1.66, 6.84)	3.07 (1.71, 7.91)	2.87 (1.60, 6.73)	0.33
<1 yr	23 (11.9)	6 (9.0)	17 (13.5)	
1-1.9 yrs	41 (21.2)	15 (22.4)	26 (20.6)	
2-4.9 yrs	64 (33.2)	21 (31.3)	43 (34.1)	
5+ yrs	65 (33.7)	25 (37.3)	40 (31.8)	0.72
Median WAZ (IQR)^a^	-2.30 (-3.46, -1.37)	-2.55 (-3.92, -1.80)	-2.10 (-3.22, -1.26)	0.02
Underweight	89 (58.9)	34 (70.8)	55 (53.4)	0.04
Median HAZ (IQR)	-3.15 (-4.30, -1.91)	-4.86 (-6.26, -3.15)	-3.02 (-4.20, -1.90)	0.10
Stunted	69 (71.9)	7 (87.5)	62 (70.5)	0.30
Median CD4% (IQR)	16.3 (11.5, 20.1)	13.6 (9.7, 16.6)	17.4 (12.9, 20.5)	0.01
Severe immunodeficiency (%)^b^	95 (59.8)	26 (70.3)	69 (56.6)	0.14

ART: antiretroviral treatment; IQR: interquartile range; PMTCT: prevention of mother-to-child transmission; WAZ: weight for age z-score; HAZ: height for age z-score;

^a^among children <10 years of age

^b^defined by age according to the 2006 WHO guidelines

The initial ART regimen was D4T/3TC/NVP for 40.5% of children. Other regimens included AZT/3TC/EFV (24.3%), D4T/3TC/EFV (18.9%), and AZT/3TC/NVP (13.5%). An additional three children received a regimen including ABC (2.0%) and one child received a regimen including emtricitabine and tenofovir (0.5%).

Children on ART experienced good immunologic recovery, with median CD4^+ ^T-cell percentage increasing to 28.9% (IQR: 22.6, 37.2), 32.4% (IQR: 25.1, 39.1), and 34.2% (IQR: 30.6, 38.7) 6, 12 and 24 months after ART initiation, respectively.

### Weight-for-age z-scores after ART initiation

**Figure 1:**
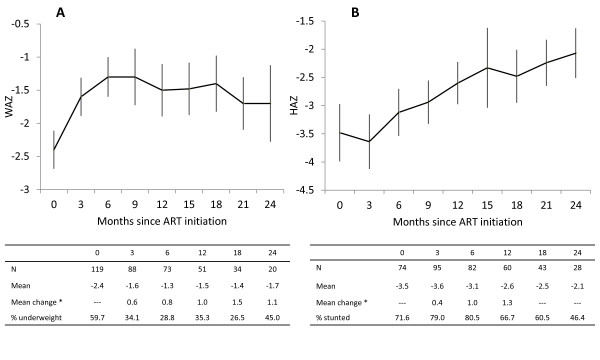
**Mean (95% CI) weight-for-age (A) and height-for-age (B) z-scores by time since ART initiation**. *sample sizes are smaller due to missing data at ART initiation

For WAZ, 128 children younger than 10 years were included in the analysis, of whom 4 (3.1%) died and 4 (3.1%) were lost to follow-up after ART initiation. WAZ increased during the first 6 months of treatment and then stabilized. Mean WAZ increased from -2.4 at treatment initiation to -1.3, -1.5, -1.4 and -1.7 at 6, 12, 18 and 24 months on ART, respectively (Figure 1A). Consequently, the proportion of underweight children decreased from 59.7% at treatment initiation to 28.8%, 35.3%, 26.5% and 45.0% at 6, 12, 18, and 24 months on ART, respectively. Results of the crude longitudinal models indicated that WAZ increased by 0.12 units per month in the first 6 months of ART, and remained stable thereafter (Table 2). Male sex, double orphan status and older age at ART initiation were significantly associated with lower WAZ. Severe immunodeficiency at ART initiation was marginally associated with lower WAZ. Differing patterns of improvement were found only by WAZ at ART initiation, with underweight children experiencing greater increases in WAZ in the first 6 months of ART.

**Table 2:** Results of the longitudinal data analysis for WAZ and HAZ after ART initiation

	***Weight for age z-scores***^***a***^		*Height for age z-scores*	
	**Coefficient (SE)**	**p-value/p-value interaction**	**Coefficient (SE)**	**p-value/p-value interaction**

***Crude model***				
Time (per month) on ART			0.053 (0.012)	<0.0001
0-6 months	0.12 (0.02)	<0.0001		
>6 months	-0.003 (0.012)	0.81		
***Adjusted model***				
Time (per month) on ART			0.10 (0.01)	<0.0001
0-6 months	0.12 (0.02)	<0.0001		
>6 months	0.003 (0.01)	0.72		
Female	0.48 (0.21)	0.02	0.60 (0.29)	0.04
Severe immunodeficiency^b, c^	-0.35 (0.21)	0.09	-0.55 (0.29)	0.06
Age (years)^c^				
0-1.9	ref		ref	
2-4.9	-0.55 (0.26)	0.04	0.49 (0.41)	0.23
≥5	-0.52 (0.27)	0.05	1.16 (0.36)	0.001
Double orphan^c^	-0.70 (0.29)	0.02		
Underweight^c^			-1.68 (0.31)	<0.0001
***Stratified by WAZ at ART initiation***^***d***^:				
WAZ ≥-2 (ref)				
0-6 months (per month)	0.04 (0.02)	0.08		
>6 months (per month)	0.04 (0.01)	0.005		
WAZ <-2 (underweight)				
0-6 months (per month)	0.19 (0.03)	<0.0001/<0.0001		
>6 months (per month)	-0.01 (0.01)	0.21/0.003		
***Stratified by age (years) at ART initiation***^***d***^:				
0-1.9 (ref): time (per month)			0.15 (0.025)	<0.0001
2-4.9: time (per month)			0.11 (0.020)	<0.0001/0.18
≥5: time (per month)			0.049 (0.014)	<0.0001/<0.0001

WAZ: weight for age z-score; HAZ: height for age z-score; SE: standard error; ART: antiretroviral treatment

^a^among children < 10 years of age

^b^defined by age according to WHO 2006 guidelines

^c^measured at ART initiation

^d^WAZ model adjusted for sex, double orphan status, severe immunodeficiency and age at ART initiation; HAZ model adjusted for sex, severe immunodeficiency and underweight at ART initiation

### Height-for-age z-scores after ART initiation

For HAZ, 152 children were included in the analysis, of whom 4 (2.6%) died and 4 (2.6%) were lost to follow-up after ART initiation. A linear increase in HAZ was observed throughout treatment and mean HAZ increased from -3.5 at treatment initiation to -3.1, -2.6, -2.5, and -2.1 at 6, 12, 18 and 24 months on ART, respectively (Figure 1B). Consequently the proportion of stunted children decreased from 71.6% at treatment initiation to 80.5%, 66.7%, 60.5%, and 46.4% at 6, 12, 18 and 24 months on ART, respectively. Results of the crude longitudinal model indicated that HAZ increased by 0.053 units per month after ART initiation (Table 2). Underweight children at ART initiation had significantly lower HAZ, while older children and females had significantly higher HAZ throughout treatment. Severe immunodeficiency at ART initiation was marginally associated with lower HAZ. Significant differences in the trajectories of HAZ were found only by age at ART initiation, with children older than 5 years at initiation experiencing significantly smaller increases in HAZ per month compared to children younger than 2 years of age.

## Discussion

In this study of young HIV-infected children in rural Zambia with good immunologic recovery on ART, both weight and height-for-age improved after initiation of ART. Age and undernutrition at ART initiation impacted both WAZ and HAZ, and differences in the trajectories of WAZ and HAZ were associated with undernutrition and age at ART initiation, respectively.

Improvements in WAZ and HAZ among HIV-infected children treated with ART were found in other studies throughout sub-Saharan Africa [7-18]. The trajectories for WAZ and HAZ after ART initiation, however, differed in this study. WAZ improved for the first 6 months and then stabilized with only minimal improvements thereafter, whereas HAZ consistently improved over time. Similar trajectories for WAZ and HAZ were reported in one study in South Africa [10], while other studies found linear improvements in WAZ during the first 24 months of treatment [11,26]. Reasons for these differences are unknown but may be due to the higher levels of undernutrition observed in this rural population [26]. Over half of the study population was underweight and three-quarters stunted at ART initiation. Differences in trajectories were found between children who were underweight and those with normal weight, with greater weight improvements in the first 6 months for children underweight at ART initiation. A more consistent increase was found for children with normal weight. Consequently, it is possible that this group of rural children experienced different trajectories than the urban populations in previous studies.

Due to the relatively young age of the study population, the impact of age at ART initiation on both WAZ and HAZ could be evaluated. Older age was associated with both WAZ and HAZ at ART initiation; however, only age impacted the trajectories for HAZ, with children older than 5 years experiencing less improvement. In other studies, HAZ did not consistently improve, with some studies finding no significant increases [12,13,17]. Discrepancies in HAZ may be due to the different age compositions of the study populations, as many studies were conducted among children with an average age older than 5 years [21]. As more infants and young children are diagnosed and started on ART, further evaluation of HAZ over time will be needed.

This study was limited by the small sample size beyond two years on ART, and the small number of children with measures available at ART initiation (Figure 1).The role of food supplementation in achieving weight and height gains in this study is unknown, as the criteria used for eligibility were not consistent across clinic staff and children did not receive supplements at every visit. In addition, no information was collected on the child's diet or on comorbidities and therefore the contribution of these factors to growth could not be assessed.

## Conclusions

This study demonstrated that rural Zambian children experienced significant improvements in both weight and height after starting ART. However, even after two years of ART approximately 25% and 50% of children remained underweight and stunted, higher than observed among HIV-negative children in the same region [27]. Consequently, successful treatment with ART was not able to fully reverse the effects of HIV on growth. Partnerships between HIV treatment and nutrition programs should be explored so that children receive an integrated care and treatment approach that includes nutritional support. Further evaluation of the impact of food supplementation on growth after ART initiation is needed.

## List of Abbreviations

3TC: lamivudine; ABC: abacavir; ART: antiretroviral therapy; AZT: zidovudine; D4T: stavudine; EFV: efavirenz; HAZ: height-for-age Z-score; IQR: interquartile range; NVP: nevirapine; PMTCT: prevention of mother-to-child transmission; SE: standard error; WAZ: weight-for-age Z-score

## Competing interests

The authors declare that they have no competing interests.

## Authors' contributions

CGS conceived of the study, performed the data analysis and participated in the writing of the manuscript. JHvD supervised the implementation of the study in Zambia and participated in the writing of the manuscript. BM was responsible for study recruitment and implementation, and reviewed the final manuscript. FH was responsible for study recruitment and implementation, and reviewed the final manuscript. PS was responsible for study recruitment and implementation, and reviewed the final manuscript. PET supervised the implementation of the study in Zambia and reviewed the final manuscript. WJM supervised the implementation of the study in the US and participated in the writing of the manuscript. All authors have read and approved the final manuscript.

## Pre-publication history

The pre-publication history for this paper can be accessed here:

http://www.biomedcentral.com/1471-2334/11/54/prepub
